# Antibacterial and antibiotic-modifying activities of three food plants (*Xanthosoma mafaffa* Lam., *Moringa oleifera* (L.) Schott and *Passiflora edulis* Sims) against multidrug-resistant (MDR) Gram-negative bacteria

**DOI:** 10.1186/s12906-016-0990-7

**Published:** 2016-01-11

**Authors:** Joachim K. Dzotam, Francesco K. Touani, Victor Kuete

**Affiliations:** Department of Biochemistry, Faculty of Science, University of Dschang, P.O. Box 67, Dschang, Cameroon

**Keywords:** Antibiotic-potentiation, Gram-negative bacteria, *Moringa oleifera*, Multi-resistance, *Passiflora edulis*, *Xanthosoma mafaffa*

## Abstract

**Background:**

The present study was designed to investigate the antibacterial activities of the methanol extract of three edible plants, namely *Xanthosoma mafaffa*, *Moringa oleifera* and *Passiflora edulis* and their synergistic effects with some commonly used antibiotics against MDR Gram-negative bacteria expressing active efflux pumps.

**Methods:**

Broth microdilution method was used to determine the minimum inhibitory concentrations (MICs) and the minimum bactericidal concentrations (MBCs) of the extracts, as well as those of antibiotics in association with the extracts.

**Results:**

The phytochemical test indicate that all tested crude extracts contained polyphenols, triterpenes and steroids whilst other phytochemical classes were selectively distributed. Extracts showed antibacterial activities with minimum inhibitory concentrations ranging from 128-1024 μg/mL on the majority of the 19 tested Gram-negative bacterial strains. Extract from the pericarp of *P. edulis* inhibited the growth of 89.5 % of the 19 tested bacterial strains, the lowest minimal inhibitory concentration (MIC) value of 128 μg/mL being recorded against *Escherichia coli* AG100 strain. In the presence of Phenylalanine-Arginine *β-*Naphtylamide (PAβN)], an efflux pump inhibitor (EPI), the activity of the extract from *X. mafaffa* increased on 40 % of tested strains. In combination with antibiotics, extracts of *X. mafaffa*, *M. oleifera* and pericarp of *P. edulis* showed synergistic effects with some antibiotics against more than 75 % of the tested bacteria.

**Conclusion:**

The results of the present study indicate that the tested plants may be used in the treatment of bacterial infections including the multi-resistant bacteria.

## Background

Infectious diseases remain the major cause of mortality amongst children and young adults worldwide, with higher prevalence in developing countries [1]. Despite the abundance of antibiotics used in chemotherapy, there is a drastic increase of resistant bacteria. Resistance to antibiotics occurs typically as a result of drug inactivation or modification, target alteration, or reduced accumulation associated with decreased permeability and⁄or increased efflux [2]. The scarcity of new antimicrobials active against MDR bacteria propels the search of chemotherapeutic agents. While 25–50 % of current pharmaceuticals are derived from natural products, it was reported that none are used as antimicrobials [3]. Investigation of substances which can potentiate the activity of commonly used antibiotics are also being intensified [4–7]. Previous studies documented the good antimicrobial potential of natural products from higher plants [8, 9]. Several food plants were also documented as potential candidate to fight MDR Gram-negative bacteria. Some of them include *Dichrostachys glomerata, Beilschmiedia cinnamomea*, *Aframomum citratum*, *Piper capense*, *Echinops giganteus*, *Fagara xanthoxyloïdes* and *Olax subscorpioïdea* [4], *Lactuca sativa, Sechium edule, Cucurbita pepo and Solanum nigrum* [10]*, Piper nigrum* and *Vernonia amygdalina* [11], *Beilschmiedia obscura, Pachypodanthium staudtii* and *Peperomia fernandopoiana* [12], *Capsicum frutescens* [13]. In our continous search of functional food plants, we designed the present work to investigate in vitro antibacterial activity of the methanol extracts of three Cameroonian food plants, *Moringa oleifera* Lam. (Moringaceae), *Xanthosoma mafaffa* (L.) Schott (Araceae), *Passiflora edulis* Sims (Passifloraceae) against MDR bacteria. The study was extended to the ability of the studied extracts to potentiate the activity of some commonly used antibiotics against some of the tested MDR bacteria.

## Methods

### Plant material and extraction

The three food plants used in this work were purchased from the Bafoussam markets (West Region of Cameroon) in January 2014. The collected plant material were the leaves of *Xanthosoma mafaffa*, *Moringa oleifera* and the fruits of *Passiflora edulis*. The plants were identified at the National herbarium (Yaounde, Cameroon) where voucher specimens were deposited under the reference numbers (Table 1). Each plant sample was air dried and then powdered. The obtained powder (200 g) was extracted with methanol (MeOH; 1 L) for 48 h at room temperature with momentary shaking. Methanol was then removed under reduced pressure to give residues which constituted the crude extract. All extracts were then kept at 4 °C until further use.

**Table 1:** Information on plants used in the present study

Species (family); Voucher number^a^	Traditional uses	Parts used traditionally	Bioactive or potentially bioactive components	Bioactivity of crude extract
*Xanthosoma mafaffa* (L.) Schott (Araceae); 18675/SRF/Cam	Bone disease (osteoporosis) [37]	Leaves and tubers	-	-
*Passiflora edulis* Sims (Passifloraceae); 65104/HNC	Anxiety, insomnia and nervousnes, antifungal, anti-inflammatory, antihypertensive [38], gastric trouble [39], antioxidant, cancer [40]	Leaves, fruit, bark and roots	ionone-I, ionone-II, megastigma-5,8-dien-4–1, megastigma-5,8(*Z*)-diene-4–1, 4,4*a*-Epoxy-4, 4*a*-dihydroedulan, 3-hydroxyedulan, edulan-I, edulan-II, passifloric acid methyl ester [40]	Methanol extract : *Sa, Sf, Bs, Ec, Pv* and *St* [40]
*Moringa oleifera* Lam. (Moringaceae); 49178/HNC	Dental caries, syphilis, typhoid, diarrhea, epilepsy, purgative, prostate cancer, water purification [41], fever, HIV-AIDS [42]	Leaves, flowers, seeds and barks	4-(4’-*O*-acetyl-*α*-*L*-rhamnopyranosyloxy)benzylisothiocyanate, 4-(-*L*-rhamnopyranosyloxy)benzylisothiocyanate, niazimicin, pterygospermin, benzylisothiocyanate and 4-(*α*-*L*-rhamnopyranosyloxy)benzylglucosinolate [43]	Aqueous and ethanol extracts of seeds against *Sa, Vc, Ec, Se, Lv* and *On* [44]

^a^(*HNC*) Herbier National du Cameroun, (*SRF*/*Cam*) Société des Réserves Forestières du Cameroun; (-): nor reported; *Sa: Staphylococcus aureus; Vc: Vibrio cholerae; Ec: Escherichia coli; Se: Salmonella enteretidis; Lv: Litopenaeus vannmaei; On: Oreochromis nicoticus; Bs: Bacillus subtilis; St: Salmonella typhi; Sf: Streptococcus faecalis; Pv: Proteus vulgaris; HIV-AIDS: Human Immunodeficiency Virus- Acquired Immuno Deficiency Syndrome*

### Preliminary phytochemical screening

The major phytochemical classes such as alkaloids (Dragendorff’s and Mayer’s tests), triterpenes (Libermann Burchard’s test), flavonoids (Aluminum chloride test), anthraquinones (Borntrager’s test), polyphenols (Ferric chloride test), sterols (Salkowski’s test), coumarins (Lacton test), saponins (Foam test) and tannins (Gelatin test) (Table 2) were investigated according to the commonly described phytochemical methods [14–17].

**Table 2:** Extraction yields and phytochemical composition of the plant extracts

Extracts	*Xanthosoma mafaffa* leaves extact	*Passiflora edulis* Pericarps (fruits) extract	*Moringa oleifera* leaves extract
Yield^a^ (%)	4.30	3.92	3.95
Alkaloids	-	-	+
Polyphenols	+	+	+
Flavonoids	-	+	+
Anthraquinones	-	-	+
Coumarins	+	-	+
Tannins	+	-	+
Triterpenes	+	+	+
Sterols	+	+	+
Saponins	+	+	+

(-): Absent; (+): Present; ^a^yield calculated as the ratio of the mass of the obtained methanol extract/mass of the plant powder

### Antimicrobial assays

#### Chemicals for antimicrobial assay

Tetracycline (TET), cefepime (CEP), ciprofloxacin (CIP), norfloxacin (NOR), chloramphenicol (CHL), ampicillin (AMP), erythromycin (ERY), kanamycin (KAN) (Sigma-Aldrich, St Quentin Fallavier, France) were used as reference antibiotics (RA). *p*-Iodonitrotetrazolium chloride (INT; Sigma-Aldrich) and Phenylalanine-Arginine-ß-Naphthylamide (PAßN; Sigma-Aldrich) were used as microbial growth indicator and efflux pumps inhibitor (EPI) respectively [18, 19].

### Microbial strains and culture media

The studied microorganisms included sensitive and resistant strains of *Escherichia coli* (ATTC8739, AG100, AG100A, AG102, AG100ATet, W3110), *Enterobacter aerogenes* (ATCC13048, EA289, EA27, EA298, CM64), *Klebsiella pneumoniae* (ATCC11296, KP55, KP63, K24), *Pseudomonas aeruginosa* (PA01, PA124), *Providencia stuartii* (ATCC29914, NEA16) obtained clinically or from the American Type Culture Collection (ATCC). Their resistance profiles have been previously reported [7, 13, 20]. Nutrient agar were used for the activation of the tested Gram-negative bacteria [21].

### INT colorimetric assay for MIC and MBC determinations

The MIC determinations on the tested bacteria were conducted using rapid *p*-iodonitrotetrazolium chloride (INT) colorimetric assay according to described methods [18] with some modifications [22, 23]. The test samples and RA were first of all dissolved in DMSO/Mueller Hinton Broth (MHB) or DMSO/7H9 broth. The final concentration of DMSO was lower than 2.5 % and does not affect the microbial growth [24, 25]. The solution obtained was then added to Mueller Hinton Broth, and serially diluted two fold (in a 96- wells microplate). One hundred microlitre (100 μL) of inoculum 1.5 x 10^6^ CFU/mL prepared in appropriate broth was then added [22, 23]. The plates were covered with a sterile plate sealer, then agitated to mix the contents of the wells using a plate shaker and incubated at 37 °C for 18 h. The assay was repeated thrice. Wells containing adequate broth, 100 μL of inoculum and DMSO to a final concentration of 2.5 % served as negative control. The MIC of samples was detected after 18 h incubation at 37 °C, following addition (40 μL) of 0.2 mg/mL of INT and incubation at 37 °C for 30 min. Viable bacteria reduced the yellow dye to a pink. MIC was defined as the lowest sample concentration that prevented the color change of the medium and exhibited complete inhibition of microbial growth [18]. The MBC was determined by adding 50 μL aliquots of the preparations, which did not show any growth after incubation during MIC assays, to 150 μL of adequate broth. These preparations were incubated at 37 °C for 48 h. The MBC was regarded as the lowest concentration of extract, which did not produce a color change after addition of INT as mentioned above [22, 23].

To evaluate the role of efflux pumps in the susceptibility of Gram-negative bacteria to *Xanthosoma mafaffa*, *Moringa oleifera* and *Passiflora edulis*, crude extracts were tested in the presence of PAßN (at 30 μg/mL) against ten selected MDR phenotypes (*E. coli* AG100, AG102 and AG100ATet, *E. aerogenes* EA289, EA27 and CM64, *K. pneumoniae* KP55 and KP63, *P. aeruginosa* PA124 and *P. stuartii* NAE16).

Extracts from *Xanthosoma mafaffa*, *Moringa oleifera* and *Passiflora edulis* were also tested in association with antibiotics at their sub-inhibitory concentrations as obtained in each bacterium (MIC/2 and MIC/4) [4, 5, 11] against ten MDR phenotypes. Fractional inhibitory concentration (FIC) was calculated as the ratio of MIC_Antibiotic in combination_/MIC_Antibiotic alone_ and the results were discussed as follows: synergy (*≤*0.5), indifferent (>0.5 to 4), or antagonism (*>*4) [26, 27]. All assays were performed in triplicate.

## Results

### Phytochemical composition

The results of the phytochemical screening (Table 2) showed that all the tested plant extracts contain polyphenols, triterpenes, sterols and saponins. The other classes of secondary metabolites were selectively distributed. Also, the extract from *M. oleifera* contains all the classes of screened secondary metabolites.

### Antibacterial activity

Results of the antibacterial activities of the tested extracts alone and in some cases in the presence of the PAβN on a panel of 19 Gram-negative bacteria are summarized in Table 3. It appears that the extracts from *P. edulis* inhibited the growth of 17/19 (89.5 %) bacteria with a concentration ranged from 128 to 1024 μg/mL. The two other samples showed selective activities, their inhibitory activity being recorded on 13/19 (68.4 %) and 11/19 (57.9 %) tested bacteria for *M. oleifera* and *X. mafaffa* extracts respectively. The lowest MIC value (128 μg/mL) was obtained with *P. edulis* and *M. oleifera* extracts on *Escherichia coli* AG100*.*

**Table 3:** Minimal Inhibitory Concentration (MIC) in μg/mL of methanol extracts from the studied plants and chloramphenicol

Bacterial strains		Tested samples, MIC and MBC and MIC in the presence of PAßN in parenthesis (μg/mL)											
		*Xanthosoma mafaffa*			*Passiflora edulis*			*Moringa oleifera*			*Chloramphenicol*		
		MIC	MBC	R	MIC	MBC	R	MIC	MBC	R	MIC	MBC	R
*E. coli*	ATCC8739	-	-	-	256	-	-	256	-	-	4	-	-
	AG100	256 (512)	-	-	128 (<4)	1024	8	128 (256)	-	-	4 (<4)	256	64
	AG100A	512	1024	2	512	1024	2	512	-	-	2	64	32
	AG102	1024 (512)	-	-	512 (1024)	1024	2	256 (1024)	-	-	8 (<4)	-	-
	AG100ATet	256 (-)	-	-	1024 (-)	-	-	1024 (-)	1024	1	64 (32)	256	4
	W3110	1024	-	-	256	-	-	256	-	-	8	16	2
*E. aerogenes*	ATCC13048	-	-	-	256	-	-	1024	-	-	8	128	16
	EA289	512 (1024)	-	-	512 (1024)	1024	2	1024 (1024)	-	-	64 (32)	512	8
	EA27	256 (16)	-	-	256 (16)	-	-	1024 (1024)	-	-	64 (<4)	512	8
	EA298	-	-	-	512	-	-	-	-	-	32	256	8
	CM64	- (-)	-	-	- (-)	-	-	- (-)	-	-	256 (4)	256	1
*K. pneumoniae*	ATCC11296	256	-	-	256	-	-	-	-	-	4	512	128
	KP55	1024 (512)	-	-	512 (1024)	-	-	256 (1024)	-	-	64 (16)	128	2
	KP63	1024 (512)	-	-	512 (1024)	-	-	- (-)	-	-	256 (32)	256	1
	K24	-	-	-	1024	-	-	-	-	-	64	256	4
*P. aeruginosa*	PA01	-	-	-	256	-	-	1024	-	-	16	256	16
	PA124	- (-)	-	-	- (-)	-	-	- (-)	-	-	64 (4)	512	8
*P. stuartii*	ATCC29914	-	-	-	512	-	-	1024	-	-	8	32	4
	NEA16	512 (1024)	-	-	256 (1024)	-	-	1024 (1024)	-	-	32 (4)	256	8

R: MIC/MBC; -: MIC > 1024 or not detected; (): values in parenthesis are MIC of substance in the presence of PAßN at 30 μg/mL

### Role of efflux pumps in the susceptibility of Gram-negative bacteria

Ten of the studied MDR bacteria were also tested for their susceptibility to the plant extracts in the presence of the PAβN at 30 μg/mL. The results showed that when combined with the extracts, PAβN improves the activity (decrease of MIC values) of *X. mafaffa* on 4/10 (40 %) of tested MDR strains. The EPI also improved the activity of *P. edulis* against *E. coli* AG100 (Table 3).

### Effects of the association of the extracts with antibiotics

A preleminary study was performed against *P. aeruginosa* PA124. This allowed selection of the appropriate sub-inhibitory concentrations of MIC/2 and MIC/4 for further studies. All the three extracts were combined separately with eight antibiotics (CIP, NOR, CHL, ERY, KAN, TET, CEF and AMP) to evaluate their possible synergetic effects. The results summarized in Tables 4, 5 and 6 showed synergic effects of the three tested extract with most of tested antibiotics except β-lactams (CEF and AMP). At MIC/2 of the extract from *X. mafaffa*, synergistic effects were observed with 6/8 (75 %) antibiotics (CIP, NOR, CHL, ERY, KAN, TET) against the tested MDR bacteria. Synergistic effects (FIC ranging from 0.5 to 0.03) were noted with the associations of each of the *X. mafaffa*, *P. edulis* and *M. oleifera* extracts and antibiotics. Low FIC values of 0.03 were obtained with the associations of *M. oleifera* extracts + ERY and *M. oleifera* extract + CHL against *Enterobacter aerogenes* EA27.

**Table 4:** MIC (FIC) of different antibiotics in association with the extract of *Xanthosoma mafaffa* at MIC/2, MIC/4 against ten MDR bacteria strains

Antibiotics	Bacterial strains, MIC (μg/mL) of antibiotics in the absence and presence of the extract											
	Extract concentration	PA124	AG100	AG102	AG100Atet	EA27	EA289	CM64	KP55	KP63	NEA16	PBSS (%)
CIP	0	16	0.50	0.50	-	1	1	0.50	2	-	1	
	CMI/2	16(1)^I^	0.50(1)^I^	**<0.50(na)** ^**S**^	-(na)	**0.25(0.25)** ^**S**^	**0.50(0.50)** ^**S**^	1(2)^I^	**<0.50(na)** ^**S**^	**16(na)** ^**S**^	**0.50(0.50)** ^**S**^	**60**
	CMI/4	16(1)^I^	0.50(1)^I^	**<0.50(na)** ^**S**^	-(na)	**0.25(0.25)** ^S^	1(1)^I^	1(2)^I^	**<0.50(na)** ^**S**^	**16(na)** ^**S**^	1(1)^I^	40
NOR	0	128	2	**1**	-	4	8	4	16	-	4	
	CMI/2	128(1)^I^	2(1)^I^	**<1(na)** ^**S**^	**128(na)** ^**S**^	8(2)^I^	16(2)^I^	32(8)^A^	16(1)^I^	128(na)^S^	8(2)^I^	30
	CMI/4	128(1)^I^	2(1)^I^	**<1(na)** ^**S**^	**128(na)** ^**S**^	32(8)^A^	16(2)^I^	32(8)^A^	16(1)^I^	-(na)	8(2)^I^	20
CHL	0	64	4	8	64	64	64	256	64	-	32	
	CMI/2	64(1)^I^	4(1)^I^	**1(0.13)** ^**S**^	**32(0.50)** ^**S**^	**4(0.06)** ^**S**^	**32(0.50)** ^S^	256(1)^I^	**16(0.25)** ^**S**^	-(na)	**16(0.50)** ^**S**^	**60**
	CMI/4	64(1)^I^	4(1)^I^	**2(0.25)** ^**S**^	64(1)^I^	**16(0.25)** ^**S**^	**32(0.50)** ^S^	256(1)^I^	**16(0.25)** ^**S**^	-(na)	**16(0.50)** ^**S**^	**50**
ERY	0	128	0.50	0.50	-	16	16	256	32	-	32	
	CMI/2	**64(0.50)** ^**S**^	**<0.50(na)** ^**S**^	**<0.50(na)** ^**S**^	**128(na)** ^**S**^	**8(0.50)** ^**S**^	**8(0.50)** ^**S**^	256(1)^I^	32(1)^I^	**128(na)** ^**S**^	**16(0.50)** ^**S**^	**80**
	CMI/4	**64(0.50)** ^**S**^	**<0.50(na)** ^**S**^	**<0.50(na)** ^**S**^	**128(na)** ^**S**^	**8(0.50)** ^**S**^	**8(0.50)** ^**S**^	256(1)^I^	64(2)^I^	**128(na)** ^**S**^	**16(0.50)** ^**S**^	**80**
KAN	0	64	0.50	0.50	2	8	32	4	16	-	16	
	CMI/2	**32(0.50)** ^**S**^	**<0.50(na)** ^**S**^	**<0.50(na)** ^**S**^	**<0.50(na)** ^**S**^	**4(0.50)** ^**S**^	**8(0.25)** ^**S**^	**2(0.50)** ^**S**^	16(1)^I^	-(na)	16(1)^I^	**70**
	CMI/4	64(1)^I^	**<0.50(na)** ^**S**^	1(2)^I^	**1(0.50)** ^**S**^	8(1)^I^	**8(0.25)** ^**S**^	4(1)^I^	16(1)^I^	-(na)	16(1)^I^	30
TET	0	16	32	2	64	32	32	8	2	8	32	
	CMI/2	**8(0.50)** ^**S**^	**16(0.50)** ^**S**^	**<0.50(na)** ^**S**^	**4(0.06)** ^**S**^	**8(0.25)** ^**S**^	**4(0.13)** ^**S**^	**2(0.25)** ^**S**^	**<0.50(na)** ^**S**^	**1(0.13)** ^**S**^	**4(0.13)** ^**S**^	**100**
	CMI/4	16(1)^I^	32(1)^I^	**1(0.50)** ^**S**^	**4(0.06)** ^**S**^	**8(0.25)** ^**S**^	**16(0.50)** ^**S**^	**2(0.25)** ^**S**^	**<0.50(na)** ^**S**^	**1(0.13)** ^**S**^	**4(0.13)** ^**S**^	**80**
AMP	0	-	-	128	-	-	-	-	-	-	-	
	CMI/2	-(na)	-(na)	128(1)^I^	-(na)	-(na)	-(na)	-(na)	**128(na)** ^**S**^	-(na)	-(na)	10
	CMI/4	-(na)	-(na)	128(1)^I^	-(na)	-(na)	-(na)	-(na)	-(na)	-(na)	-(na)	00
CEF	0	-	64	32	-	-	-	-	-	-	-	
	CMI/2	-(na)	64(1)^I^	32(1)^I^	-(na)	-(na)	-(na)	-(na)	**256(na)** ^**S**^	-(na)	-(na)	10
	CMI/4	-(na)	64(1)^I^	32(1)^I^	-(na)	-(na)	-(na)	-(na)	-(na)	-(na)	-(na)	00

^a^Antibiotics [*TET* tetracycline, *CIP* ciprofloxacin, *NOR* norfloxacin, *KAN* kanamycin, *CHL* chloramphenicol, *ERY* erythromycin, *AMP* ampicillin, *CEF* cefepime]

^b^Bacterial strains: *Escherichia coli* [AG100, AG102, AG100Atet], *Pseudomonas aeruginosa* [PA124], *Enterobacter aerogenes* [CM64, EA27, EA289], *Klebsiella pneumonia* [KP55], *Providencia stuartii* [NAE16]

^c^PBSS: percentage of bacteria strain on which synergism has been observed; (): *FIC* (Fractional Inhibitory Concentration) of the antibiotics after association with plants extract; S: Synergy, I: Indifference; na: not applicable; The values in bold represent the cases of synergy between extract and antibiotic; (-): >256 μg/mL

**Table 5:** MIC (FIC) of different antibiotics in association of the extract of *Passiflora edulis* at MIC/2, MIC/4 against ten MDR bacteria strains

Antibiotics	Bacterial strains, MIC (μg/mL) of antibiotics in the absence and presence of the extract											
	Extract concentration	PA124	AG100	AG102	AG100Atet	EA27	EA289	CM64	KP55	KP63	NEA16	PBSS (%)
CIP	0	16	0.50	0.50	-	1	1	0.50	2	-	1	
	CMI/2	16(1)^I^	0.50(1)^I^	0.50(1)^I^	-(na)	2(2)^I^	**<0.50(na)** ^**S**^	8(16)^A^	**<0.50(na)** ^**S**^	-(na)	1(1)^I^	20
	CMI/4	16(1)^I^	0.50(1)^I^	0.50(1)^I^	-(na)	4(4)^A^	1(1)^I^	64(256)^A^	**<0.50(na)** ^**S**^	-(na)	1(1)^I^	10
NOR	0	128	2	1	-	4	8	4	16	-	4	
	CMI/2	128(1)^I^	2(1)^I^	**<1(na)** ^**S**^	**128(na)** ^**S**^	4(1)^I^	**4(0.50)** ^**S**^	64(16)^A^	**4(0.25)** ^**S**^	-(na)	4(1)^I^	40
	CMI/4	128(1)^I^	2(1)^I^	**<1(na)** ^**S**^	**128(na)** ^**S**^	16(4)^I^	8(1)^I^	-(na)^A^	**4(0.25)** ^**S**^	-(na)	4(1)^I^	30
CHL	0	64	4	8	64	64	64	256	64	-	32	
	CMI/2	64(1)^I^	**2(0.50)** ^**S**^	**<0.50(na)** ^**S**^	**32(0.50)** ^**S**^	**2(0.03)** ^**S**^	**32(0.50)** ^**S**^	**128(0.50)** ^**S**^	64(1)^I^	-(na)	**16(0.50)** ^**S**^	70
	CMI/4	64(1)^I^	**2(0.50)** ^**S**^	**1(0.13)** ^**S**^	**32(0.50)** ^**S**^	**16(0.25)** ^**S**^	64(1)^I^	256(1)^I^	64(1)^I^	-(na)	**16(0.50)** ^**S**^	50
ERY	0	128	0.50	0.50	-	16	16	256	32	-	32	
	CMI/2	**64(0.50)** ^**S**^	**<0.50(na)** ^**S**^	**<0.50(na)** ^**S**^	-(na)	128(8)^A^	**1(0.06)** ^**S**^	256(1)^I^	**8(0.25)** ^**S**^	**128(na)** ^**S**^	**16(0.50)** ^**S**^	**70**
	CMI/4	128(1)^I^	<0.50(na)^S^	**<0.50(na)** ^**S**^	-(na)	128(8)^A^	16(1)^I^	256(1)^I^	**2(0.06)** ^**S**^	**128(na)** ^**S**^	**16(0.50)** ^**S**^	**50**
KAN	0	64	0.50	0.50	2	8	32	4	16	-	16	
	CMI/2	64(1)^I^	**<0.50(na)** ^**S**^	0.50(1)^I^	**<0.50(na)** ^**S**^	**4(0.50)** ^**S**^	**2(0.06)** ^**S**^	128(32)^A^	**2(0.13)** ^**S**^	-(na)	16(1)^I^	**60**
	CMI/4	64(1)^I^	**<0.50(na)** ^**S**^	0.50(1)^I^	**1(0.50)** ^**S**^	**4(0.50)** ^**S**^	**8(0.25)** ^**S**^	-(na)	**2(0.13)** ^**S**^	-(na)	16(1)^I^	**60**
TET	0	16	32	2	64	32	32	8	2	8	32	
	CMI/2	**8(0.50)** ^**S**^	**16(0.50)** ^**S**^	**<0.50(na)** ^**S**^	**8(0.13)** ^**S**^	64(2)^I^	**2(0.06)** ^**S**^	32(4)^A^	**<0.50(na)** ^**S**^	**4(0.50)** ^**S**^	**8(0.25)** ^**S**^	**80**
	CMI/4	16(1)^I^	**16(0.50)** ^**S**^	**<0.50(na)** ^**S**^	**8(0.13)** ^**S**^	64(2)^I^	**4(0.13)** ^**S**^	64(8)^A^	**<0.50(na)** ^**S**^	**4(0.50)** ^**S**^	**8(0.25)** ^**S**^	**70**
AMP	0	-	-	128	-	-	-	-	-	-	-	
	CMI/2	-(na)	-(na)	**<1(na)** ^**S**^	-(na)	-(na)	-(na)	-(na)	-(na)	**256(na)** ^**S**^	-(na)	20
	CMI/4	-(na)	-(na)	**32(0.25)** ^**S**^	-(na)	-(na)	-(na)	-(na)	-(na)	**256(na)** ^**S**^	-(na)	20
CEF	0	-	64	32	-	-	-	-	-	-	-	
	CMI/2	-(na)	128(2)^I^	**8(0.25)** ^**S**^	-(na)	-(na)	-(na)	-(na)	-(na)	**128(na)** ^**S**^	-(na)	20
	CMI/4	-(na)	128(2)^I^	32(1)^I^	-(na)	-(na)	-(na)	-(na)	-(na)	**128(na)** ^**S**^	-(na)	10

^a^Antibiotics [*TET* tetracycline, *CIP* ciprofloxacin, *NOR* norfloxacin, *KAN* kanamycin, *CHL* chloramphenicol, *ERY* erythromycin, *AMP* ampicillin, *CEF* cefepime]

^b^Bacterial strains: *Escherichia coli* [AG100, AG102, AG100Atet], *Pseudomonas aeruginosa* [PA124], *Enterobacter aerogenes* [CM64, EA27, EA289], *Klebsiella pneumonia* [KP55], *Providencia stuartii* [NAE16].

^c^PBSS: percentage of bacteria strain on which synergism has been observed; (): *FIC* (Fractional Inhibitory Concentration) of the antibiotics after association with plants extract; S: Synergy, I: Indifference; na: not applicable; The values in bold represent the cases of synergy between extract and antibiotic; (-): >256 μg/mL

**Table 6:** MIC (FIC) of different antibiotics after the association of the extract of *Moringa oleifera* at MIC/2, MIC/4 against ten MDR bacteria strains

Antibiotics	Bacterial strains, MIC (μg/mL) of antibiotics in the absence and presence of the extract											
	Extract concentration	PA124	AG100	AG102	AG100Atet	EA27	EA289	CM64	KP55	KP63	NEA16	PBSS (%)
CIP	0	16	0.50	0.50	-	1	1	0.50	2	-	1	
	CMI/2	32(2)^I^	0.50(1)^I^	0.50(1)^I^	-(na)	1(1)^I^	**0.50(0.50)** ^**S**^	4(8)^A^	**<0.50(na)** ^**S**^	-(na)	1(1)^I^	20
	CMI/4	32(2)^I^	0.50(1)^I^	0.50(1)^I^	-(na)	1(1)^I^	**0.50(0.50)** ^**S**^	8(16)^A^	**<0.50(na)** ^**S**^	-(na)	1(1)^I^	20
NOR	0	128	2	1	-	4	8	4	16	-	4	
	CMI/2	128(1)^I^	2(1)^I^	**<1(na)** ^**S**^	-(na)	4(1)^I^	**4(0.50)** ^**S**^	64(16)^A^	**8(0.50)** ^**S**^	-(na)	**2(0.50)** ^**S**^	40
	CMI/4	128(1)^I^	2(1)^I^	**<1(na)** ^**S**^	-(na)	4(1)^I^	**4(0.50)** ^**S**^	128(32)^A^	16(1)^I^	-(na)	**2(0.50)** ^**S**^	30
CHL	0	64	4	8	64	64	64	256	64	-	32	
	CMI/2	**32(0.50)** ^**S**^	4(1)^I^	**1(0.13)** ^**S**^	64(1)^I^	**32(0.50)** ^**S**^	**32(0.50)** ^S^	**128(0.50)** ^**S**^	**2(0.03)** ^**S**^	-(na)	32(1)^I^	**60**
	CMI/4	64(1)^I^	4(1)^I^	**2(0.25)** ^**S**^	64(1)^I^	**32(0.50)** ^**S**^	**32(0.50)** ^**S**^	256(1)^I^	**4(0.06)** ^**S**^	-(na)	32(1)^I^	40
ERY	0	128	0.50	0.50	-	16	16	256	32	-	32	
	CMI/2	128(1)^I^	**<0.50(na)** ^**S**^	**<0.50(na)** ^**S**^	**128(na)** ^**S**^	**8(0.50)** ^**S**^	**8(0.50)** ^**S**^	**128(0.50)** ^**S**^	**1(0.03)** ^**S**^	**128(na)** ^**S**^	**16(0.50)** ^**S**^	**90**
	CMI/4	128(1)^I^	**<0.50(na)** ^**S**^	**<0.50(na)** ^**S**^	**128(na)** ^**S**^	**8(0.50)** ^**S**^	**8(0.50)** ^**S**^	256(1)^I^	**4(0.13)** ^**S**^	**128(na)** ^**S**^	**16(0.50)** ^**S**^	**80**
KAN	0	64	0.50	0.50	2	8	32	4	16	-	16	
	CMI/2	**32(0.50)** ^**S**^	**<0.50(na)** ^**S**^	**<0.50(na)** ^**S**^	**<0.50(na)** ^**S**^	**1(0.13)** ^**S**^	**2(0.06)** ^**S**^	8(2)^I^	**4(0.25)** ^**S**^	-(na)	**4(0.25)** ^**S**^	**80**
	CMI/4	64(1)^I^	**<0.50(na)** ^**S**^	**<0.50(na)** ^**S**^	**1(0.50)** ^**S**^	**2(0.25)** ^**S**^	**4(0.13)** ^**S**^	256(64)^A^	**8(0.50)** ^**S**^	-(na)	**8(0.50)** ^**S**^	**70**
TET	0	16	32	2	64	32	32	8	2	8	32	
	CMI/2	16(1)^I^	**16(0.50)** ^**S**^	**1(0.50)** ^**S**^	**8(0.13)** ^**S**^	**16(0.50)** ^**S**^	**4(0.13)** ^**S**^	8(1)^I^	**<0.50(na)** ^**S**^	**4(0.50)** ^**S**^	**8(0.25)** ^**S**^	**80**
	CMI/4	16(1)^I^	32(1)^I^	**1(0.50)** ^**S**^	**8(0.13)** ^**S**^	**16(0.50)** ^**S**^	**4(0.13)** ^**S**^	64(8)^A^	**<0.50(na)** ^**S**^	**4(0.50)** ^**S**^	**8(0.25)** ^**S**^	**70**
AMP	0	-	-	128	-	-	-	-	-	-	-	
	CMI/2	-(na)	-(na)	128(1)^I^	-(na)	-(na)	-(na)	-(na)	-(na)	-(na)	-(na)	00
	CMI/4	-(na)	-(na)	128(1)^I^	-(na)	-(na)	-(na)	-(na)	-(na)	-(na)	-(na)	00
CEF	0	-	64	32	-	-	-	-	-	-	-	
	CMI/2	-(na)	64(1)^I^	32(1)^I^	-(na)	-(na)	-(na)	-(na)	-(na)	-(na)	-(na)	00
	CMI/4	-(na)	64(1)^I^	32(1)^I^	-(na)	-(na)	-(na)	-(na)	-(na)	-(na)	-(na)	00

^a^Antibiotics [*TET* tetracycline, *CIP* ciprofloxacin, *NOR* norfloxacin, *KAN* kanamycin, *CHL* chloramphenicol, *ERY* erythromycin, *AMP* ampicillin, CEF: cefepime]

^b^Bacterial strains: *Escherichia coli* [AG100, AG102, AG100Atet], *Pseudomonas aeruginosa* [PA124], *Enterobacter aerogenes* [CM64, EA27, EA289], *Klebsiella pneumonia* [KP55], *Providencia stuartii* [NAE16]

^c^PBSS: percentage of bacteria strain on which synergism has been observed; (): *FIC* (Fractional Inhibitory Concentration) of the antibiotics after association with plants extract; S: Synergy, I: Indifference; na: not applicable; The values in bold represent the cases of synergy between extract and antibiotic; (-): >256 μg/mL

## Discussion

Phytochemical screening revealed the presence of several classes of secondary metabolites such as alkaloids, polyphenols, flavonoids, anthraquinones, coumarins, saponins, tannins, triterpenes and steroids. Several molecules belonging to these classes were found to be active on pathogenic microorganisms [3, 28–30]. The presence of such metabolites in the studied plant extracts can provide a preliminary explanation on their antibacterial activities. Differences were observed in the antibacterial activities of the extracts. These could be due to the differences in their chemical composition as well as in the mechanism of action of their bioactive constituents [3]. As shown in Table 2, all the extracts are rich in secondary metabolites especially the extract from *M. oleifera* (which contains all the tested classes); However, activity does not depend only on the presence of secondary metabolites in the plant extracts, but mostly on their concentration and the possible interactions with other constituents.

To the best of our knowledge, the antibacterial activity of *X. mafaffa* is being reported here for the first time. The inhibitory activity of *M. oleifera* was previously reported against some bacteria such as *Escherichia coli*, *Pseudomaonas aeruginosa*, *Stapylococcus aureus* and *Salmonella typhii* [31]. The present study confirmed the antimicrobial potential of this plant and provide additional information on it ability to inhibit the growth of MDR bacteria.

The results of this work are very important taking in account the medicinal importance of the tested MDR bacteria [32–36] and also the fact that samples used are edible plants. In the presence of PAβN (EPI), the antibacterial activity of some of the extracts increased, suggesting that some active constituents may have intracellular target. In the presence of the EPI, the activity of *M. oleifera* remain unchanged, indicating that the bioactive compounds of this extract are not the substrates of bacterial efflux pumps, as the tested MDR bacteria over-express efflux pumps [32–36]. However, it should be observed that in certain cases (Table 3), MIC values of *Xanthosoma mafaffa* and *Passiflora edulis* extracts increased in the presence of PAßN. A possible explanation is that some active constituents of these extracts may act in the cell coat, inhibiting the synthesis of peptidoglycan. In such case, in the absence of PAßN, such compounds are extruded from the cytoplasm of bacteria by efflux pumps, then re-cross the cell membrane to reach their target in the coat, explaining while the MIC value is lower; in presence of PAßN, the efflux pumps are blocked and such compounds could not be expelled from the cytoplasm, reducing their concentration in the cell coat and consequently their activity, explaining their higher MIC values.

In the recent years, scientists intensified the search of substances with ability to restore the activity of available antibiotics to MDR bacteria. In this work, synergistic effects were noted with the associations of *X. mafaffa*, *P. edulis* and *M. oleifera* extracts and some antibiotics, providing additional information of their possible use to combat MDR phenotypes.

## Conclusion

The results of the present investigation show that *X. mafaffa*, *P. edulis* and *M. oleifera* may be useful in the control of many infectious diseases, particularly those caused by the multidrug resistant Gram-negative bacteria. These extracts may be used alone or in combination with certain antibiotics such as tetracycline, ciprofloxacin, norfloxacin, chloramphenicol, erythromycin, kanamycin but not beta-lactamines. The isolation of the active compounds from the three plants constitutes the limitation of the present study and will be further performed. Also, further investigations of the plant extracts are warranted in vivo to validate their use for the control of infectious diseases.

## Abbreviations

AMP: Ampicillin
ATCC: American type culture collection
CEF: Cefepime
CFU: Colony forming unit
CHL: Chloramphenicol
CIP: Ciprofloxacin
DMSO: Dimethylsulfoxyde
EPI: Efflux pump inhibitor
ERY: Erythromycin
FIC: Fractional inhibitory concentration
INT: p-iodonitrotetrazolium chloride
KAN: Kanamycin
MDR: Multidrug resistant
MHB: Mueller hinton broth
MIC: Minimal inhibitory concentration
NOR: Norfloxacin
PAßN: Phenylalanine arginine-ß-Naphthylamide
RND: Resistance nodulation-cell division
STR: Streptomycin
TET: Tetracycline
